# Association of the *STAT4*, *CDKN1A*, and *IRF5* variants with risk of lupus nephritis and renal biopsy classification in patients in Vietnam

**DOI:** 10.1002/mgg3.1648

**Published:** 2021-03-09

**Authors:** Trung Dung Nghiem, Gia Tuyen Do, Long Hoang Luong, Quy Linh Nguyen, Ha Viet Dang, Anh Nguyen Viet, Thuy Thu Nguyen, Van Khanh Tran, Thanh Van Ta, Thinh Huy Tran

**Affiliations:** ^1^ Nephro‐Urology Department Bach Mai Hospital Hanoi Vietnam; ^2^ Center for Gene and Protein Research Hanoi Medical University Hanoi Vietnam

**Keywords:** *CDKN1A*, *IRF5*, risk factor, SLEDAI, *STAT4*, systemic lupus erythematous

## Abstract

**Background:**

Lupus nephritis is a common complication of systemic lupus erythematosus (SLE, OMIM #15200) in the Asian population and a main contributor to mortality and morbidity. In this study, we evaluate the variants on three genes *STAT4*, *CDKN1A*, and *IRF5* and their association with lupus nephritis.

**Method:**

One hundred fifty‐two SLE patients with confirmed lupus nephritis (through biopsy) and 76 healthy controls were recruited. Genotyping of SNPs on three gene *STAT4*, *CDKN1A*, and *IRF5*, phenotypic, and laboratory assessment were performed; renal biopsy and classification were carried out for the patient group.

**Results:**

Carriers of rs7582694 C alleles on *STAT4* have higher risk of lupus nephritis (OR 2.0; 95% CI [1.14, 3.19]; *p* = 0.015), at higher risk of hematuria and higher serum level of dsDNA antibodies compared to controls (*p* < 0.05) and were more likely to have nephrotic histopathology grading of class III or higher. No association was observed for *CDKN1A*; and no variation was observed for the *IRF5* gene in both the study and control group.

**Conclusion:**

This study investigates the relationship between *STAT4*, *CDKN1A*, and *IRF5* gene and SLE in a Vietnamese patient population. Patients with the C allele (*STAT4*) in rs7582694 were associated with a more severe disease phenotype.

## Graphical abstract

Despite the current evidence of genetic factor on the risk of Lupus Nephritis, there are currently no study assessing these variants in a Vietnamese population, and only a few studies have investigated the genotype‐phenotype correlation in Lupus Nephritis patients. Thus, in this report, we evaluate the polymorphisms of these three genes (STAT4, CDKN1A and IRF5) and their association with the phenotype of patients with Lupus nephritis. In which the disease progression was assessed with renal biopsy and careful clinical work‐up.

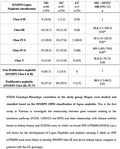

## 1 INTRODUCTION

Systemic lupus erythematosus (SLE, OMIM #15200) is a heterogeneous systemic autoimmune disease characterized by multiple organ pathology with complex genetic components (Mok & Lau, 2003). The estimated prevalence of SLE in the Asia‐Pacific region ranges from 4.3 to 45.3 per 100,000, with Asian having a higher observed rate of lupus nephritis and related mortality (Jakes et al., 2012; Lech & Anders, 2013; Yap & Chan, 2015). SLE etiology involves both genetic and environmental factors. The disease also follows a polygenic disease model, with different genes implicated for different populations. Systemic lupus erythematosus with nephritis (lupus nephritis) occurs in 40%–75% of SLE patients and is a major contributor to morbidity and mortality (Doria et al., 2006). To date, the etiology and pathogenesis of Lupus nephritis are still not fully understood.

The role of cytokines and their signaling pathways have been investigated in the pathogenicity as well as disease severity of SLE and lupus nephritis. Out of the factors studied, interferon pathway and its components have been established to have a significant contribution to the risk of developing SLE. Increased production of type I interferon (IFN) and expression of IFN‐inducible genes are commonly observed in SLE and may be crucial in the molecular pathogenesis of renal involvement in SLE (Crow, 2014). IRF5 (interferon regulatory factors 5) are transcriptional factor regulating interferon expression and have been implicated in the risk of developing of SLE in various population (Cham et al., 2012). Graham et al. (2007) found functional variants on the IRF5 gene to be responsible for altered risk of developing SLE (Graham et al., 2007). Another gene, *CDKN1A*, has been suggested to play a role in mediating the action of IFNα and INFγ. Kim et al. (2009) found that a regulatory SNP at position −899 of the CDKN1A gene is associated with an increased risk of developing SLE and lupus nephritis (Kim et al., 2009).

Signal transducer and activator of transcription factor 4 (*STAT4*) are also related to the interferon‐signaling pathway in which it is mediated through IFNα and, in turn, activates the transcription of IFNγ. A study on multiple populations has found single nucleotide polymorphism of *STAT4* to be moderately correlated to the risk of SLE (Namjou et al., 2009). Another study by Sigurdsson et al. (2008) found that 10/53 variants in *STAT4* gene were correlated to the risk of SLE, and the SNPs with highest level of association were rs10181656 and rs7582694 (Sigurdsson et al., 2008).

Despite the evidence of these genetic factor on the risk of SLE, there are currently no study assessing these variants in a Vietnamese population, and only a few studies have investigated the genotype–phenotype correlation in patients with systemic lupus erythematous. Thus, in this report, we evaluate the polymorphisms of these three genes (*STAT4*, *CDKN1A*, and *IRF5*) and their association with the phenotype of patients with lupus nephritis.

## 2 METHOD

This is a case‐control study conducted at the Department of Nephrology, Bach Mai hospital, Hanoi, Vietnam, from January 2014 to February 2017. A total of 152 SLE patients with confirmed lupus nephritis and 76 healthy controls were recruited for this study. The study abides by the Declaration of Helsinki in regards to study involving human subjects. The study protocol was approved by the Ethic Committee of Bach Mai Hospital. Informed consents were obtained from all participants.

### 2.1 Patients and controls

Patient's diagnosis and selection were based on the SLICC (Systemic Lupus International Collaborating Clinics Classification Criteria) criteria for systemic lupus erythematosus (Petri et al., 2012). Disease activity was assessed based on the SELENA‐SLEDAI scoring system (SLE modified Disease Activity Index; Bombardier et al., 1992). The participant was assessed for parameters including complete blood count, erythrocyte sediment rate, C3, C4, antinuclear antibodies, and anti‐dsDNA. Renal biopsy was performed for the patient group. The ISN/RPS (2003) classification was used for histopathology grading of lupus nephritis (Weening et al., 2004). Exclusion criteria include age <15 years old, clinical indication of other autoimmune diseases, patients on dialysis, or contraindication for renal biopsy.

### 2.2 Genetic analysis

Upon enrollment, blood samples were obtained from each subject and collected into sterile EDTA tubes for genotyping and laboratory analysis. Genomic DNA was isolated from 2‐ml whole blood sample obtained from each subject using the Wizard^®^ Genomic DNA Purification Kit (Promega). DNA was stored at −80°C until the time of analysis. Using the PCR‐RFLP technique, we amplified and genotyped rs7582694 in *STAT4* gene; rs762624 in *CDKN1A* gene; rs6953165; rs2004640, and rs41298401 in intron 1 of the *IRF5* gene were identified using Sanger's sequencing. The primers and restriction enzyme used in the study are provided in the Supporting Information.

### 2.3 Statistical analysis

Statistical analyses were performed with STATA version 14.0. SLE patient and control groups were tested for conformity to Hardy–Weinberg equilibrium (HWE) by Pearson's χ^2^ test. The genotype distribution and allele frequency were compared using either χ^2^ or Fisher exact tests, as appropriate. The odds ratios (ORs) were calculated as 95% confidence interval (95% CI) using logistic regression analyses to evaluate the independent effect of *STAT4*, *IRF5*, and *CDKN1A* different genotypes on clinical manifestations of SLE, SELENA‐SLEDAI. Continuous parameters were compared by independent sample *t* test or analysis of variance test. Bonferroni's correction for multiple comparisons was applied. *p* value less than 0.05 was considered significant.

## 3 RESULT

The patient group consists of 152 patients diagnosed with SLE with lupus nephritis complication; mean age was 29.8 (±9.1), mean age at diagnosis was 28.2 (±9.4), and male/female ratio was 14/138. The mean disease duration was 15.2 ± 29.3 months. The control group was match for gender and age with mean age 32.1 ± 9.4 and male/female ratio of 7/69. The demographic of the two groups and clinical and biochemical parameter of the patient group are presented in Table 1.

**TABLE 1:** Patient and control group demographic and clinical and laboratory result at admission

Characteristic	Patient group *N* = 152	Control group *N* = 76	*p*
Age, years, mean ± SD	29.8 ± 9.1	32.1 ± 9.4	0.08
Gender, male/female	14/138	7/69	1
Age of diagnosis	28.2 ± 9.4	NA	
Disease duration, months	15.2 ± 29.3	_	
Photosensitivity, *n* (%)	72 (47.4)	_	
Malar rash, *n* (%)	85 (55.9)	_	
Discoid rash, *n* (%)	0	_	
Alopecia, *n* (%)	56 (36.8)	_	
Oral ulcer, *n* (%)	6 (4)	_	
Arthritis, *n* (%)	28 (18.4)	_	
Hemolytic anemia, *n* (%)	130 (85.5)	_	
Thrombocytopenia, *n* (%)	5 (3.3)	_	
Leukopenia, *n* (%)	15 (9.9)	_	
Lymphopenia, *n* (%)	68 (44.7)	_	
Positive ANA, *n* (%)	142 (93.4)	_	
Positive anti‐dsDNA, *n* (%)	98 (64.5)	_	
Decreased C3, *n* (%)	136 (89.5)	_	
Decreased C4, *n* (%)	84 (55.3)	_	
Serositis, *n* (%)	97 (63.8)	_	
Seizure/Psychosis, *n* (%)	6 (4)	_	
SELENA‐SLEDAI score	18.0 ± 5.6	_	

### 3.1 Results of genetic analysis

Distribution of the *STAT4* and *SKDN1A* genotype is displayed in Table 2. The results of our study show that there was a significant difference in distribution of *STAT4* genotype at position rs7582694 between SLE patients lupus nephritis and controls.

**TABLE 2:** Genotype distribution of the case/control groups

Genotype	Patient group *N* = 152	Control group N = 76	OR (95% CI)	*p*
*CDKN1A*				
rs762624				
CC	44 (28.9%)	24 (31.6%)	1	
AC	81 (53.3%)	38 (50.0%)	1.16 (0.62, 2.19)	0.64
AA	27 (17.8%)	14 (18.4%)	1.05 (0.46, 2.39)	0.9
AC + AA	108 (71.1%)	52 (68.4%)	1.13 (0.62, 2.06)	0.68
*STAT4*				
rs7582694				
GG	60 (39.5%)	43 (56.6%)	1	
CG	87 (57.2%)	32 (42.1%)	1.95 (1.11, 3.42)	0.02^*^
CC	5 (3.3%)	1 (1.3%)	3.58 (0.40, 31.8)	0.25
CG + CC	92 (60.5%)	33 (43.4%)	2.0 (1.14, 3.49)	0.015^*^

* The difference is statistically significant with *p* < 0.05.

Specifically, the distribution of patients/controls genotypes of rs7582694 was as follows: GG genotype 39.5%/56.6%; CG 57.2%/42.1%; CC 3.3%/1.3%. The carriers of C alleles on *STAT4* had higher risk of lupus nephritis (OR 2.0; 95% CI [1.14, 3.19]; *p* = 0.015). In our study, patients with STAT4 rs7582694 C/C or G/C genotypes were at higher risk of hematuria and had higher serum level of dsDNA antibodies (*p* < 0.05).

No difference was observed in the genotype distribution between patients with lupus nephritis and control group in the *CDKN1A* gene position SNP rs762624. *IRF5* genotype distribution at three sites, namely, SNP rs6953165, rs2004640, and rs41298401 between patients with lupus nephritis and the control group was similar. All three SNPs in both groups showed a homozygous inheritance pattern. No correlation was observed between the SELENA‐SLEDAI score and the genotype of the patients.

Patients with lupus nephritis carrying C alleles on *STAT4*, position rs7582694 were at higher risk of class III kidney injury (OR 11.4); 13 times more likely to develop class IV‐S and 8.9 times to develop renal lesion class IV‐G compared with patients without the allele, the differences were statistically significant with *p* < 0.05 (Table 3).

**TABLE 3:** *STAT4* genotype–phenotype correlation in the study group

ISN/RPS lupus nephritis classification	GG *n* = 60 *n* (%)	GC *n* = 87 *n* (%)	CC *n* = 5 *n* (%)	GG–GC/CC OR (95% CI) *p*
Class I + II	6 (10.0)	1 (1.2)	0 (0)	1
Class III	10 (16.7)	19 (21.8)	0 (0)	11.4 (1.2, 108.3) 0.03^*^
Class IV‐S	12 (20.0)	24 (27.6)	2 (40.0)	13 (1.41, 120.3) 0.02^*^
Class IV‐G	23 (38.3)	31 (35.6)	3 (60)	8.9 (1.001, 78.6) 0.05^*^
Class V	0 (15.0)	12 (13.8)	0 (0.0)	8 (0.81, 78.73) 0.08
Nonproliferative nephritis (ISN/RPS Classes I and II)	6 (85.7)	1 (14.3)	0	1
Proliferative nephritis (ISN/RPS Classes III, IV, and V)	54 (37.2)	86 (59.3)	5 (3.5)	10.1 (1.2, 86.2) 0.03

Biopsy were studied and classified based on the ISN/RPS (2003) classification of lupus nephritis.

* The difference is statistically significant with *p* < 0.05.

## 4 DISCUSSION

Functionally, *STAT4* is the main transcriptional regulatory molecule for IL‐12 and, as such, is pivotal to the development of a fully functioning T‐helper 1 immune response. In addition, *STAT4* transmits signals from the receptors of IL‐12 and IL‐23 and can therefore contribute to autoimmune responses by affecting the functions of several innate and adaptive immune cells. Thus, it has gathered interest in study of risk factors contributing to SLE. Remmers et al. (2007) have demonstrated association of one *STAT4* SNP (rs7574865) with SLE in Europeans. Lee et al. (2007) replicated the association of *STAT4* with rheumatoid arthritis in European and Korean patients. Three SNPs (rs10181656, rs13017460, and rs1517352) were significantly associated with rheumatoid arthritis in Korean patients. Also, Korman et al. (2008) reported an association with rs7574865 and primary Sjögren's syndrome (PSS) in a study of 124 Caucasian PSS subjects and 1143 controls (*p* = 0.01). PSS and SLE share overlapping autoantibody profiles (such as anti‐Ro) and B lymphocyte hyperactivity, supporting the notion that related autoimmune diseases share common risk variants in *STAT4*. However, previous study only investigated the relationship between *STAT4* SNPs and SLE. This is, to our knowledge, the first study to elucidate the relationship between *STAT4* and lupus nephritis, as well as investigating the degree of genotype–phenotype correlation in these patients.

By taking renal biopsy and doing histological assessment, combining with genetics profile of the patients, we can accurately discern the genotype‐phenotype association between these immunologic gene variants and the degree of nephrotic syndrome. The 2003 International Society of Nephrology (ISN)/Renal Pathology Society (RPS) Classification of lupus nephritis was designed to eliminate ambiguities and standardize definitions regarding the classification of lupus nephritis. Allele C in rs7582694 of *STAT4* has a moderate correlation with risk of lupus nephritis (OR =2.0; *p* = 0.015); however, it has a very strong association with higher lupus nephritis grading (10.1 times more likely to develop class III or higher ISN/RPS histology grading). Thus, we speculate that genetic profiling could also serve as a prognostic factor for clinical use and possibly for better management and prevention of complication in the patients.

Despite the finding of Kim et al. (2009) that the patients carrying AA or AC genotypes are 1.43 times more likely to develop SLE versus the CC group (*p* = 0.00047), we were unable to find an association between CDKN1A SNP rs762624 and risk of developing lupus nephritis. We were able to observe a different inheritance pattern in regard to the *IRF5* gene, all 3 SNPs studied showed 100% homozygous for one allele; thus, they were not informative for the analysis.

To our knowledge, this is the first study to investigate the genetics profile of the three immune‐related gene *STAT4*, *IRF5*, *CDKN1A* in Vietnam and the first to focus on lupus nephritis patient. We used phenotypic profiling with SLEDAI to assess disease activity and ISN/RPS to standardize histology grading of renal biopsies.

## 5 CONCLUSION

This is the first study in Vietnam to investigate the relationship between gene variants relating to the interferon pathway (*STAT4*, *CDKN1A*, and *IRF5*) and their relationship with disease activity based on kidney biopsy and SLEDAI score, in which we found SNP rs7582694 (STAT4) was a risk factor for the development of lupus nephritis and patients carrying C allele on SNP rs7582694 were more likely to develop ISN/RPS class III and above kidney injury compare to patients with the GG genotype. Patients with C alleles were also more likely to have high anti‐dsDNA antibody and hematuria.

## CONFLICT OF INTERESTS

The authors declare no conflict of interest.

## AUTHOR CONTRIBUTIONS

Trung Dung Nghiem and Gia Tuyen Do contributed equally to this work. Trung Dung Nghiem, Gia Tuyen Do, Long Hoang Luong, and Thinh Huy Tran conceived and designed the study and analysis. Quy Linh Nguyen, Ha Nguyen Viet, and Anh Nguyen Viet contributed in data collection and carried out the experiments. Anh Nguyen Viet, Thuy Thi Nguyen, Van Khanh Tran, and Thanh Van Ta performed analysis and finalized the results. Long Hoang Luong and Trung Dung Nghiem contributed in drafting of the manuscript. All authors have read and approved the final version for publication.

## ETHIC APPROVAL AND CONSENT TO PARTICIPATE

The study design was reviewed and approved by the Ethical board of Hanoi Medical University. The study complies with the Declaration of Helsinki regarding the use of human samples and identifiable information. Informed consent was obtained from the patients regarding the use of the samples and information for research purpose.

## CONSENT TO PUBLISH

The patients gave consent to publish the patients’ information including clinical and genetics information. No identifiable information was disclosed in any form.

## Supporting information

Table S1‐S4

## Data Availability

The datasets used and/or analyzed during the current study are available from the corresponding author on reasonable request. We however cannot provide personal information or data contain identification of the patients in any form.
