# Supplementary material for: Association of the STAT4, CDKN1A, and IRF5 variants with risk of lupus nephritis and renal biopsy classification in patients in Vietnam
Source: Mol Genet Genomic Med. 2021 Mar 9;9(4):e1648. doi: 10.1002/mgg3.1648 (PMC8123735; doi:10.1002/mgg3.1648)
Supplement: Supplementary file 1 — Table S1‐S4 [file MGG3-9-e1648-s001.docx]

**SUPPLEMENTARY INFORMATION**

**Table 1:** Association between *STAT4 genotype* and NIH-AI (National Institute of Health - Activity Index) score (Bajema et al., 2018)

| **NIH-AI index** | **GG**  (n=60)  n (%) | **GC**  (n=87)  n (%) | **CC**  (n=5)  n (%) | **GG – GC/CC**  OR (95%CI)  p |
| --- | --- | --- | --- | --- |
| Low  (0-7 point) | 39  (65,0) | 50  (57,5) | 3  (60,0) | 1 |
| Medium  (8-11 point) | 18  (30,0) | 25  (28,7) | 1  (20,0) | 1,1 (0,5 – 2,2)  0,8 |
| High  (12-24 point) | 3  (5,0) | 12  (13,8) | 1 (20,0) | 3,2 (0,9 – 12,0)  0,09 |
| Mean ± SD | 5,8 ± 4,1 | 6,6 ± 4,4 | 7,0 ± 4,2 |  |

**Table 2:** Association between *STAT4 genotype* and NIH-CI (National Institute of Health - Chronicity Index) score (Bajema et al., 2018)

| **NIH-CI Index** | **GG**  (n=60)  n (%) | **GC**  (n=87)  n (%) | **CC**  (n=5)  n (%) | **GG – GC/CC**  OR (95%CI)  p |
| --- | --- | --- | --- | --- |
| Low  (≤1 point) | 34  (56,7) | 46  (52,9) | 1  (20,0) | 1 |
| Medium  (2-3 point) | 18  (30,0) | 23  (26,4) | 0 | 0,9 (0,4 – 2,0)  0,8 |
| High  (≥4 point) | 8  (13,3) | 18  (20,7) | 4  (80,0) | 2,0 (0,8 – 5,0)  0,1 |
| Mean ± SD | 1,7 ± 2,1 | 2,1 ± 2,0 | 4,8 ± 3,2 |  |

**Table 3:** Result summary of different population study on *STAT4* association with SLE

| **No** | **Title** | **Year** | **Population** | **Design** | **Results** | **Citation** |
| --- | --- | --- | --- | --- | --- | --- |
| ***** | This study:  Association Of The STAT4, CDKN1A and IRF5 Variants with Risk of Lupus Nephritis and Renal Biopsy Classification in Patients in Vietnam. |  | Vietnamese | Descriptive with case-control: 152 Lupus nephritis patients and 76 healthy controls | Carriers of rs7582694 C alleles on STAT4 have higher risk of Lupus Nephritis (OR 2.0; 95% CI: 1.14-3.19; p=0.015), at higher risk of hematuria and higher serum level of dsDNA antibodies compared to controls (p < 0.05) and were more likely to have nephrotic histopathology grading of class III or higher. |  |
| 1 | Role of STAT4 Polymorphisms in Systemic Lupus Erythematosus in a Japanese Population: A Case-Control Association Study of the STAT1-STAT4 Region | 2008 | Japanese in Tokyo, Japan | Descriptive with case-control  308 SLE patients and 306 controls  - The American College of Rheumatology criteria for SLE | In the first screening, rs7574865, rs11889341, and rs10168266 in STAT4 were most significantly associated (P < 0.01).  The same STAT4 risk allele is associated with SLE in Caucasian and Japanese populations. The contribution of STAT4 for the genetic background of SLE may be greater in the Japanese population than in Americans of European descent. | (Kawasaki et al., 2008) |
| 2 | Association of STAT4 Polymorphism With Severe Renal Insufficiency in Lupus Nephritis:  To analyze single nucleotide polymorphisms (SNPs) for association with lupus nephritis, its severe form proliferative nephritis and renal outcome, in two Swedish cohorts | 2013 | Swedish in clinics in Sweden. | Cohort I: 567 SLE, 512 controls  Cohort II: 145 SLE, 619 controls  Standards:  - 1982 ACR criteria for SLE  - WHO classification system for LN | In the case-control analysis of cohort I, four highly linked SNPs in STAT4 were associated with lupus nephritis with genome wide significance with p = 3.7×10−9, OR 2.20 for the best SNP rs11889341.  In the case-only meta-analysis of the two cohorts, the STAT4 SNP rs7582694 was associated with severe renal insufficiency with p  = 1.6×10−3 and OR 2.22. | (Bolin et al., 2013) |
| 3 | Association of Genetic Variations in the STAT4 and IRF7/KIAA1542 Regions With Systemic Lupus Erythematosus in a Northern Han Chinese Population  To investigate the association of single-nucleotide polymorphisms (SNPs) in the IRF7/KIAA1542 region (rs4963128, rs2246614, and rs702966) and in STAT4 (rs7574865 and rs7582694) with SLE disease in a Northern Han Chinese population | 2013 | Northern Han Chinese population. | 748 patients and 750 healthy controls. | A strong association between rs7574865 (odds ratio = 0.68; 95% confidence interval 0.59–0.79; p = 1.57 × 10−6) and SLE and between rs7574865 and the production of anti-Sm antibodies. Additionally, rs4963128 and rs2246614 were correlated with a variety of clinical subphenotypes, such as lupus nephritis, arthritis, and the production of anti-SSA/B autoantibodies, despite a lack of significant association between these two SNPs and SLE disease susceptibility in general. | (Li et al., 2011) |
| 4 | Population Differences in SLE Susceptibility Genes: STAT4 and BLK, but Not PXK, Are Associated With Systemic Lupus Erythematosus in Hong Kong Chinese  we compared the association of several newly discovered susceptibility genes for systemic lupus erythematosus (SLE) between populations of European origin and two Asian populations | 2009 |  | 910 SLE patients and 1440 healthy controls from Chinese living in Hong Kong, and 278 SLE patients and 383 controls in Thailand | Data confirmed association of STAT4 (rs7574865, odds ratio (OR) =1.71, P=3.55 x 10(-23)) and BLK (rs13277113, OR=0.77, P=1.34 x 10(-5)) with SLE. It was showed that rs7574865 of STAT4 is also linked to hematologic disorders and potentially some other subphenotypes of the disease. More than one genetic variant in STAT4 were found to be associated with the disease independently in our populations (rs7601754, OR=0.59, P=1.39 x 10(-9), and P=0.00034 when controlling the effect of rs7574865). | (Yang et al., 2009) |

**Table 4:** List of primer and restriction enzyme used in the study


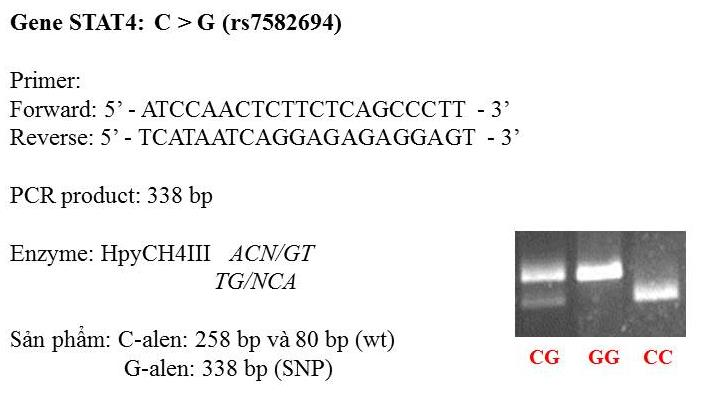


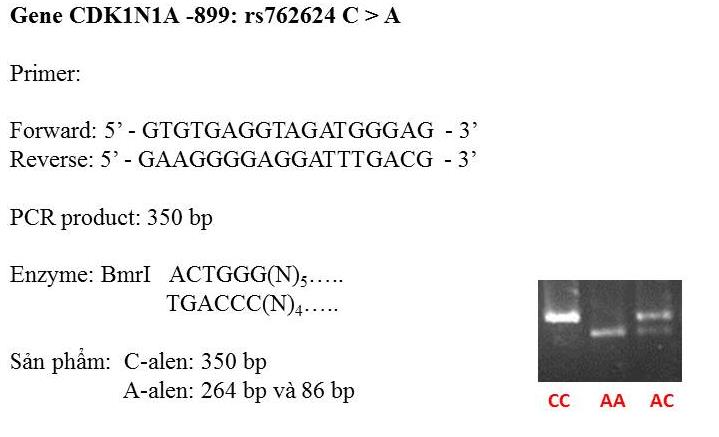


**
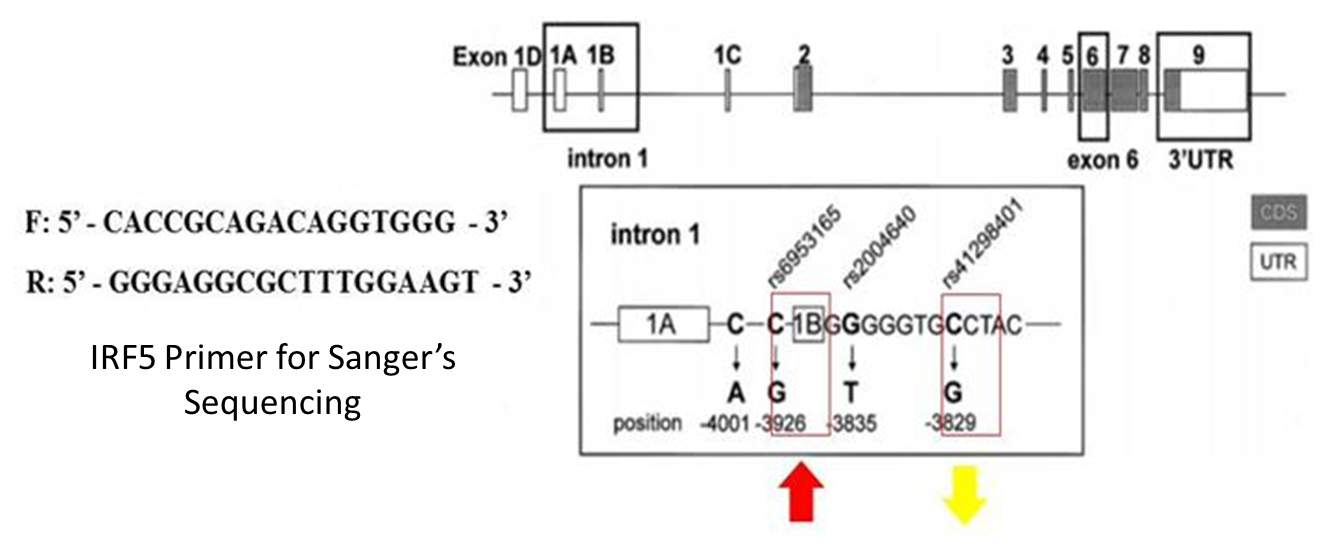
**

**REFERENCES**

Bajema, I. M., Wilhelmus, S., Alpers, C. E., Bruijn, J. A., Colvin, R. B., Cook, H. T., D’Agati, V. D., Ferrario, F., Haas, M., Jennette, J. C., Joh, K., Nast, C. C., Noël, L.-H., Rijnink, E. C., Roberts, I. S. D., Seshan, S. V., Sethi, S., & Fogo, A. B. (2018). Revision of the International Society of Nephrology/Renal Pathology Society classification for lupus nephritis: Clarification of definitions, and modified National Institutes of Health activity and chronicity indices. *Kidney International*, *93*(4), 789–796. https://doi.org/10.1016/j.kint.2017.11.023

Bolin, K., Sandling, J. K., Zickert, A., Jönsen, A., Sjöwall, C., Svenungsson, E., Bengtsson, A. A., Eloranta, M.-L., Rönnblom, L., Syvänen, A.-C., Gunnarsson, I., & Nordmark, G. (2013). Association of STAT4 Polymorphism with Severe Renal Insufficiency in Lupus Nephritis. *PLoS ONE*, *8*(12). https://doi.org/10.1371/journal.pone.0084450

Kawasaki, A., Ito, I., Hikami, K., Ohashi, J., Hayashi, T., Goto, D., Matsumoto, I., Ito, S., Tsutsumi, A., Koga, M., Arinami, T., Graham, R. R., Hom, G., Takasaki, Y., Hashimoto, H., Behrens, T. W., Sumida, T., & Tsuchiya, N. (2008). Role of STAT4 polymorphisms in systemic lupus erythematosus in a Japanese population: A case-control association study of the STAT1-STAT4 region. *Arthritis Research & Therapy*, *10*(5), R113. https://doi.org/10.1186/ar2516

Li, P., Cao, C., Luan, H., Li, C., Hu, C., Zhang, S., Zeng, X., Zhang, F., Zeng, C., & Li, Y. (2011). Association of genetic variations in the STAT4 and IRF7/KIAA1542 regions with systemic lupus erythematosus in a Northern Han Chinese population. *Human Immunology*, *72*(3), 249–255. https://doi.org/10.1016/j.humimm.2010.12.011

Su, Y., Zhao, Y., Liu, X., Guo, J., Jiang, Q., Liu, X., Zhang, F., Zheng, Y., Li, X., Song, H., Huang, C., Huang, Y., Wang, T., Pan, S., Li, C., Liu, X., Zhu, L., Zhang, C., & Li, Z. (2010). Variation inSTAT4is associated with systemic lupus erythematosus in Chinese Northern Han population. *Chinese Medical Journal*, *123*(22), 3173–3177. https://doi.org/10.3760/cma.j.issn.0366-6999.2010.22.001

Yang, W., Ng, P., Zhao, M., Hirankarn, N., Lau, C. S., Mok, C. C., Chan, T. M., Wong, R. W. S., Lee, K. W., Mok, M. Y., Wong, S. N., Avihingsanon, Y., Lee, T. L., Ho, M. H. K., Lee, P. P. W., Wong, W. H. S., & Lau, Y. L. (2009). Population differences in SLE susceptibility genes: STAT4 and BLK , but not PXK , are associated with systemic lupus erythematosus in Hong Kong Chinese. *Genes & Immunity*, *10*(3), 219–226. https://doi.org/10.1038/gene.2009.1
